# Prevalence and Factors Associated with Syphilis among Mothers with Missed Opportunities for Antenatal Syphilis Testing in Rural Western Uganda: A Cross-Sectional Study

**DOI:** 10.1155/2023/2971065

**Published:** 2023-08-24

**Authors:** Theoneste Hakizimana, Joy Muhumuza, Fabrice Molen Selamo, Marie Pascaline Sabine Ishimwe, Rogers Kajabwangu, Osman Mohamud Jelle, Joshua Muhumuza, Sonye Magugu Kiyaka, Sandra Nyakato, Yarine Fajardo

**Affiliations:** ^1^Department of Obstetrics and Gynecology, Kampala International University, Western Campus, Uganda; ^2^Department of Surgery, Kampala International University, Western Campus, Uganda; ^3^Faculty of General Medicine, University of Rwanda, Rwanda; ^4^Department of Laboratory, Fort Portal Regional Referral Hospital, Kabarole, Uganda

## Abstract

**Background:**

Early prenatal syphilis testing and treatment are essential preventative measures for maternal syphilis and associated adverse pregnancy outcomes of pregnancy; however, data shows that two-thirds of all cases are missed among women who visit prenatal care center at least once but are not tested for syphilis. This study determined the prevalence and factors associated with syphilis infection among mothers with missed opportunities for antenatal syphilis testing in rural western Uganda delivered at Fort Portal Regional Referral Hospital (FRRH).

**Methods:**

A cross-sectional study was done during the period from April 2022 to June 2022. A total of 124 participants had been recruited consecutively from postnatal ward of FRRH. Pretested questionnaires were used to obtain information on data required for analysis. Venous blood sampling (2 ml taken from the forearm using anticoagulant free vacutainer) was done for all mothers who missed opportunity for prenatal syphilis testing using both RPR and TPHA. Descriptive statistics followed by binary logistic regression analysis was done using SPSS version 22.0.

**Results:**

The prevalence of syphilis infection was 27 (21.8%). After adjusted analysis, having more than one sexual partners in the past one year was associated with higher odds of syphilis infection (aOR = 24.922, 95% CI: 4.462-139.201, *p* < 0.001), and staying with the partner was found to be associated with lower odds of syphilis infection (aOR = 0.213, 95% CI: 0.040-1.142, *p* = 0.050).

**Conclusions:**

The study identified high prevalence of syphilis infection among mothers with missed opportunities for antenatal syphilis testing, and this was positively associated with having more than one sexual partners in the past one year and negatively associated with not staying with partner.

## 1. Introduction

Globally, syphilis continues to be a serious public health concern, affecting an estimated 12 million individuals each year [1], the majority of whom live in underdeveloped countries [2]. The prevalence of syphilis infection among pregnant mothers in sub-Saharan Africa is estimated to be 2.7 percent, putting approximately one million pregnancies at risk each year [3]. The high prevalence rates are ranging from 17.4% in Cameroon to 8.4% in South Africa, 4% in Uganda, and 2.5 percent in Burkina Faso [4]. In 2016, the WHO delivered the specific strategies for syphilis testing and treatment for pregnant mothers, published in 2017, which recommended syphilis screening for all pregnant mothers at first antenatal contact [5], in the third trimester for diagnosis of new infections acquired during pregnancy, and at the time of delivery for women who missed prenatal testing to enable early detection and treatment of infections in women and neonates [6].

While mother-to-child transmission (MTCT) of syphilis is connected to a lack of antenatal care, WHO data shows that the majority of unfavorable pregnancy outcomes caused by maternal syphilis occur in women who received antenatal care but were not appropriately screened or treated [7]. In a study done in New York City, 68 pregnant women were associated with cases of congenital syphilis, among which 47 pregnant women who received prompt antenatal care did not receive an initial syphilis test until 45 days before delivery [8]. In Africa and worldwide, there is scarcity of data on the proportion of syphilis-seropositive mothers among those who missed prenatal testing. The study done in Tanzania recruited 663 women, and only 49.9% were screened for syphilis during the antenatal visit, among those who did not test for syphilis at ANC 6 (1.8%) were positive for syphilis [6]. In the same study, the overall prevalence of syphilis at delivery among pregnant women who did not test or tested negative for syphilis at ANC visits was 2.3%.

In a study done in South Africa, the seroconversion rate for syphilis at the time of delivery was 2.7%, the seropositivity among those who did not test at ANC was 18.2%, and the total prevalence of syphilis at delivery among those who tested and those who were not tested in ANC was 9.3% [9]. A study conducted in the Mwanza Region of Tanzania showed that 1809 participants were not tested for syphilis prenatally, in which 144 (8.9%) became syphilis positive at delivery [10].

In regard to the factors leading to seropositivity of syphilis, the study done in Ethiopia showed that participants who were HIV positive had a significantly higher prevalence of syphilis than those who were HIV negative. Furthermore, in this study, the use of condoms, marital status, and maternal age were not statistically significant risk factors for syphilis [11]. In a similar study, the knowledge about use of condoms to prevent STDs and a prior history of multiple sexual encounters were linked to lower rates of syphilis infection [12]. Unpublished data from a study conducted between March 2021 and May 2021 revealed that, of the 1252 mothers who gave birth at the FRRH, 50.3% did not have documented results of syphilis screening in their antenatal cards and were therefore treated as syphilis negative. Therefore, this study determined prevalence and factors associated with syphilis infection among mothers with missed opportunities for antenatal syphilis testing in rural western Uganda delivered at FRRH.

## 2. Methods

### 2.1. Data Collection

A cross-sectional study was done from postnatal ward of FRRH. A total of 124 mothers were consecutively enrolled from April 2022 to June 2022. Fort Portal Regional Referral Hospital is a public and teaching hospital for both undergraduate and postgraduate students of Kampala International University and other tertiary institutions around. It is situated in Fort Portal town in Kabarole District, around 300 kilometers west of Kampala, the country's capital city. 350 beds are available for inpatient care at FRRH, with 105 of those beds located in the Department of Obstetrics and Gynecology. The medical facility has a modern, accredited laboratory equipped to conduct syphilis tests. The study participants were from catchment areas that included the districts of Kabarole, Bundibugyo, Kamwenge, Kasese, Ntoroko, and Kyenjojo. Participants in this study were postpartum mothers (at least 12 hours postdelivery) enrolled consecutively as long as they had antenatal cards with them showing lack of prenatal syphilis testing. A standardized pretested questionnaire developed in both English and Rutooro (the local language) was used to gather information on factors associated with syphilis seropositivity until we reached the target sample size. All qualified mothers were asked for their consent. At the time of recruitment, mothers without prenatal cards were excluded from the study.

### 2.2. Syphilis Testing

Venous blood sampling (2 ml taken from the forearm using anticoagulant free vacutainer) was done for all mothers who missed opportunity for prenatal syphilis testing, and rapid plasma reagin (RPR) was carried out. Samples which were seropositive for syphilis were retested using Treponema pallidum hemagglutination assay (TPHA) to confirm active syphilis infection. Both RPR and TPHA were conducted within one hour after blood sampling.

### 2.3. Sample Size Determination

Calculation of sample size was done using the Kish Leslie formula (1965):

(1) $$\begin{array}{c} n=\frac{z^{2}p\left(1-p\right)}{e^{2}}, \end{array}$$

where *n* is the estimated minimum required sample size, *p* is the proportion of a characteristic in a sample (mothers not tested for syphilis during antenatal period), *e* is the margin of error set at 5%, and *z* is 1.96 (for 95% confidence interval).

Using *p* = 8.9%, the proportion of syphilis seropositive mothers among those who did not test for syphilis during antenatal care in a study done in tanzania, Mwanza region [13]

(2) $$\begin{array}{c} n=\frac{{\left(1.96\right)}^{2}\times 0.089\times \left(1-0.089\right)}{{\left(0.05\right)}^{2}}=124.5, \end{array}$$

where *n* is 124 participants.

### 2.4. Data Analysis

The dataset was done using Microsoft Excel version 16 and coded and loaded into SPSS version 22.0 for analysis. The proportion of syphilis-seropositive mothers was calculated as number of mothers with positive RPR and TPHA out of all mothers enrolled into the study and expressed as frequency and percentages. The factors associated with syphilis seropositivity among mothers with missed opportunity for antenatal syphilis testing were determined using binary logistic regression. A bivariate analysis was performed using cross-tabulation at 95% confidence interval (CI) to assess the likely effect. Maternal factors that were found with a *p* ≤ 0.05 and others with biological plausibility were considered to a multivariate analysis at 95% confidence interval (CI) to remove the confounding factors. Factors that turned up with *p* ≤ 0.05 were considered significant in this analysis. The odds ratio, confidence interval, and *p* value were used to interpret and display the results of both bivariate and multivariate analyses.

## 3. Results

### 3.1. Characteristics of the Study Participants

A total of 124 mothers in postnatal ward were enrolled with a response rate of 100%. The majority of study participants were found between 20 and 29 years of age (73, 58.9%), multiparous (63, 50.8%), married (97, 78.2%), from rural areas (87, 70.2%), and staying in <5 km from health facility (79, 63.7%) and have primary education level (70, 56.3%). 122 (98.4%) of the participants have attended antenatal care, 93 (75%) reported no history of abortion, and 97 (78.2%) reported no history of other sexually transmitted diseases (STDs). The majority of respondents were HIV negative (109, 87.9%), while 85 (68.5%) of our participants were staying with their partners and 94 (75.8%) have had more than one sexual partners in the past one year. The majority of mothers have ever heard of syphilis (84, 67.7%) (Table 1).

### 3.2. Prevalence of Syphilis Infection among Mothers with Missed Opportunities for Antenatal Syphilis Testing Delivered at FRRH

**Figure 1:**
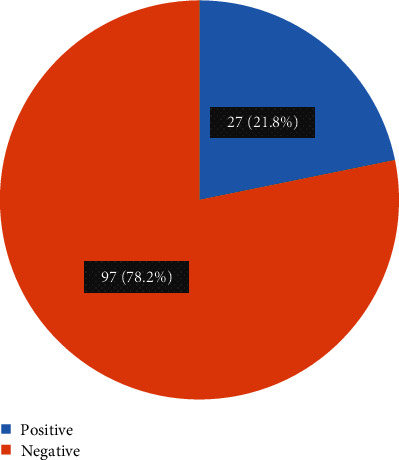
Prevalence of syphilis infection among mothers with missed opportunities for syphilis testing at FRRH.

Of 124 participants recruited in this study, the overall prevalence of syphilis infection among mothers with missed opportunity for antenatal syphilis testing was 27 (21.8%). However, the majority were negative for syphilis infection (97, 78.2%) as shown in Figure 1.

### 3.3. Factors Associated with Syphilis Infection among Mothers with Missed Opportunities for Antenatal Syphilis Testing

This study revealed that the number of sexual partners in the past 1 year and staying with partner became independently associated with syphilis infection among mothers with missed opportunities for antenatal syphilis testing. Precisely, mothers who reported to have had more than 1 sexual partners in the past one year were 25 times more likely to have syphilis infection (aOR = 24.922, 95% CI: 4.462-139.201, *p* < 0.001). Mothers who were not staying with their partners were 0.2 times less likely to have syphilis infection (aOR = 0.213, 95% CI: 0.040-1.142, *p* = 0.05) (Table 1).

## 4. Discussion

In this study, it was found that 21.8% of 124 respondents who did not test for syphilis in the prenatal period became syphilis seropositive. This reflects the prevalence of syphilis-seropositive mothers among those who missed the opportunity for syphilis testing. This high prevalence implies the lack of opportunity for timely diagnosis and treatment of syphilis, putting the pregnancy at potential risk including congenital syphilis, which may impede the WHO goal to achieve less than 50 occurrences of congenital syphilis per 100 000 live births in 80% of the target countries by 2030 [14].

The prevalence of 21.8% obtained in this study is consistent with 18.2% obtained in South Africa [9]. This prevalence is much higher than 1.2% obtained in Bugando Medical Centre (BMC), Tanzania [6], and 8% obtained in the Mwanza Region of Tanzania. The difference could be explained by the fact that Kabarole District is one of the districts in Uganda which are severely affected by HIV infections, with an HIV prevalence rate of 16% which is almost 3 times the national prevalence of 5.8% in the age group of 15-49 years [15], and yet, both HIV and syphilis have the same modes of transmission. In Uganda, there is an increased burden of maternal syphilis and weak implementation of prenatal testing and treatment policies, compounded by the fact that a big percentage of mothers do not attend ANC in early pregnancy [13] as underlined by our study findings where the majority of our respondents started their ANC in the 2nd trimester of pregnancy (60.5%). The high prevalence of syphilis seropositivity obtained in this study could also be accounted by low male partner attendance at ANC seen in Uganda which impairs the closure of the loop of infection to identify and treat all potentially infected partners. The study done in Uganda showed that the overall postenrollment partner attendance in ANC was 18.3% despite partner notification [16]. There is a paucity of data on factors associated with syphilis infection among mothers who did not screen for syphilis prenatally. In regard to factors associated, it was found that mothers who had more than one sexual partners in the past 1 year were more likely to have syphilis infection. In present study, 75.8% of study participants had no multiple sexual partners in the past 1 year while 24.2% had multiple sexual partners. Among those who had multiple sexual partners, 46.7% were found to have syphilis infection. Our findings are consistent with the studies done in northwest and southern Ethiopia [17, 18]. Inconsistence was with the study done in Brazil [19] and the one done in Cameroon [11]. The difference can be accounted on difference in geographic location, study setting, and study population. In addition to that, the majority of our participants were between 20 and 29 years, and sexual activity may be higher in this age group increasing the risk of having multiple sexual activity and thus syphilis infection. The study revealed that the majority of participants have only attained primary education (57.3%) and were not sure of the mode of transmission of syphilis (52.4%); this may probably increase risk of syphilis infection due to insufficient knowledge about syphilis. In a study done in Brazil, 66.10% of participants reported to have received no information about sexually transmitted diseases (STD) like syphilis, during prenatal care [20]. Syphilis screening is recommended for all pregnant mothers at first antenatal contact [5], and high-risk pregnant women including those with multiple sexual partners should be screened again between 28 and 32 weeks into their pregnancy, as well as at delivery [21].

In this study, mothers who were not staying with their partners were less likely to have syphilis infection. In present study, 31.5% of mothers were not living with their partners of which 8.1% became syphilis seropositive. In a study done in Brazil, women who were not living with their partners were at increased risk of syphilis infection; however, this became statistically insignificant after adjusted analysis (*p* = 0.143) [19]. Syphilis is mainly acquired by sexual contact with infected mucocutaneous lesions or abraded skin [22]; hence, not staying with the partner may be protective of sexual transmitted diseases mainly if the male partner has multiple sexual partners.

### 4.1. Study Strength and Limitation

To the best of our knowledge, our study is the first documented in Uganda particularly western Uganda, and for that, the findings will serve as a baseline for future studies in the region. In this study, a nontreponemal test (rapid plasma reagin) was carried out and confirmed by a treponemal test (TPHA) for seroreactive samples to ensure the reliability of the results as per WHO recommendations. Women who did not have antenatal records but had attended ANC were excluded from the study. Risk factors for these women may be different from those who had the cards.

## 5. Conclusions

The prevalence of syphilis among mothers who missed the opportunity for prenatal syphilis testing in rural western Uganda is high as compared to other findings from studies done in East Africa, and it is more likely associated with having multiple sexual partners in the past 1 year and less likely associated with not staying with partner. We recommend sensitization of community about the importance of early testing and treatment of syphilis at ANC. ANC staff should identify and do counselling of high-risk mothers like those with multiple sexual partners for regular follow-up and syphilis testing with their partners.

## Figures and Tables

**Table 1:** Factors associated with syphilis infection among mothers with missed opportunities for antenatal syphilis testing (*N* = 124).

Variables	Categories	Syphilis test		cOR (95% CI)	*p* value	aOR (95% CI)	*p* value
		Negative (*n*, %)	Positive (*n*, %)				
Age	<20	18 (81.8)	4 (18.2)	0.538 (0.150-2.262)	0.436	0.284 (0.052-1.5557)	0.147
	20-29	58 (79.5)	15 (20.5)	0.329 (0.252-1.832)	0.679	0.364 (0.102-1.297)	0.119
	≥30	21 (72.4)	8 (27.6)	Ref		Ref	

Marital status	Single	13 (65.0)	7 (35.0)	2.211 (0.776-6.296)	0.137		
	Separated	6 (85.7)	1 (14.3)	0.684 (0.078-6.026)	0.732		
	Married	78 (80.4)	19 (19.6)	Ref			

Residence	Rural	67 (77.0)	20 (23.0)	1.279 (0.489-3.349)	0.616		
	Urban	30 (81.1)	7 (18.9)	Ref			

Level of education	Preprimary	4 (80.0)	1 (20.0)	0.75 (0.032-17.506)	0.858		
	Primary	50 (71.4)	20 (28.6)	1.2 (0.118-12.233)	0.878		
	Secondary	40 (88.9)	5 (11.1)	0.375 (0.032-4.331)	0.432		
	Tertiary	3 (75.0)	1 (25.0)	Ref			

Parity	1	33 (71.7)	13 (28.3)	1.083 (0.292-4.023)	0.905		
	2 to 4	53 (84.1)	10 (15.9)	0.519 (0.137-1.960)	0.333		
	≥5	11 (73.3)	4 (26.7)	Ref			

Distance to health facility	≥5 km	35 (77.8)	10 (22.2)	1.042 (0.430-2.523)	0.927		
	<5 km	62 (78.5)	17 (21.5)	Ref			

ANC attendance	No	1 (50.0)	1 (50.0)	3.692 (0.223-61.052)	0.361		
	Yes	96 (78.7)	26 (21.3)				

History of abortion and stillbirth	Yes	27 (87.1)	4 (12.9)	0.451 (0.1425-1.425)	0.175	0.385 (0.075-1.971)	0.252
	No	70 (75.3)	23 (24.7)	Ref		Ref	

HIV status	Positive	9 (60.0)	6 (40.0)	2.794 (0.896-8.712)	0.077	2.729 (0.707-10.533)	0.145
	Negative	88 (80.7)	21 (19.3)	Ref		Ref	

History of other STDs	Yes	21 (77.8)	6 (22.2)	1.034 (0.37-2.89)	0.949	0.997 (0.231-4.303)	0.997
	No	76 (78.4)	21 (21.6)	Ref		Ref	

Staying with partner	No	29 (74.4)	10 (25.6)	1.379 (0.564-3.372)	0.481^∗^	0.213 (0.040-1.142)	0.050^∗^
	Yes	68 (80.0)	17 (20.0)	Ref		Ref	

Number of sexual partners in the past 1 year	>1 partner	16 (53.3)	14 (46.7)	5.452 (2.16-13.762)	<0.001^∗^	24.922 (4.462-139.201)	<0.001^∗^
	1 partner	81 (86.2)	13 (13.8)	Ref		Ref	

Heard of syphilis	No	34 (85.0)	6 (15.0)	0.529 (0.195-1.437)	0.212		
	Yes	63 (75.0)	21 (25.0)	Ref			

^∗^ *p* ≤ 0.05. ANC: antenatal care; cOR: crude odds ratio; aOR: adjusted odds ratio.

## Data Availability

The dataset that was used and analyzed in this study is available from the corresponding author in case needed. Upon reasonable request, dataset used is also available to all authors with permission from Dr. Theoneste Hakizimana (email: theonestehakizimana5@gmail.com).

## Abbreviations

FRRH: Fort Portal Regional Referral Hospital
WHO: World Health Organization
ANC: Antenatal care
MTCT: Mother-to-child transmission.

## References

[1] Africa S. (2018). 2012: utility of laboratory-based information.

[2] Wu X., Hong F., Lan L., Zhang C., Feng T., Yang Y. (2016). Poor awareness of syphilis prevention and treatment knowledge among six different populations in South China. *BMC Public Health*.

[3] Hussen S., Tadesse B. T. (2019). Prevalence of syphilis among pregnant women in sub-saharan africa: a systematic review and meta-analysis.

[4] Gertrude B. N. (2009). The prevalence of syphilis and pregnancy outcome among HIV infected pregnant women attending antenatal syphilis screening program at IDI, Uganda. *Geneva Foundation for Medical Education and Research*.

[5] Trinh T., Leal A. F., Mello M. B. (2019). Syphilis management in pregnancy: a review of guideline recommendations from countries around the world. *Sexual and Reproductive Health Matters*.

[6] Lawi J. D. T., Mirambo M. M., Magoma M. (2015). Sero-conversion rate of syphilis and HIV among pregnant women attending antenatal clinic in Tanzania: a need for re-screening at delivery. *BMC Pregnancy and Childbirth*.

[7] Peeling R. W., Mabey D., Kamb M. L., Chen X.-S., Radolf J. D., Benzaken A. S. (2018). Syphilis. *Nature Reviews Disease Primers*.

[8] Slutsker J. S., Hennessy R. R., Schillinger J. A. (2018). Factors contributing to congenital syphilis cases — New York City, 2010 – 2016.

[9] Qolohle D. C., Hoosen A. A., Moodley J., Smith A. N., Mlisana K. P. (1995). Serological screening for sexually transmitted infections in pregnancy: is there any value in re-screening for HIV and syphilis at the time of delivery?. *Transmitted Infections*.

[10] Watson‐Jones D., Changalucha J., Gumodoka B. (2002). Syphilis in pregnancy in Tanzania. I. Impact of maternal syphilis on outcome of pregnancy. *The Journal of Infectious Diseases*.

[11] Halle-Ekane G. E., Ojong A. R., Bechem N., Obinchemti T. E., Halle-Ekane N. M., Nana P. N. (2018). Screening for syphilis among third trimester pregnant women in a low resource setting: a missed opportunity?. *International Journal of Tropical Disease & Health*.

[12] Tareke K., Munshea A., Nibret E. (2019). Seroprevalence of syphilis and its risk factors among pregnant women attending antenatal care at Felege Hiwot Referral Hospital, Bahir Dar, northwest Ethiopia: a cross - sectional study. *BMC Research Notes*.

[13] Oloya S., Lyczkowski D., Orikiriza P. (2020). Prevalence, associated factors and clinical features of congenital syphilis among newborns in Mbarara Hospital, Uganda. *BMC Pregnancy and Childbirth*.

[14] Kojima N., Klausner J. D. (2019). An update on the global epidemiology of syphilis. *Current Epidemiology Reports*.

[15] Ssekankya V., Githaiga S. K., Aleko T. (2021). Factors influencing utilization of HIV testing services among boda-boda riders in Kabarole District, southwestern Uganda: a cross-sectional study. *BioMed Research International*.

[16] Parkes-Ratanshi R., Kimeze J. M., Nakku-Joloba E. (2020). Low male partner attendance after syphilis screening in pregnant women leads to worse birth outcomes: the Syphilis Treatment of Partners (STOP) randomised control trial. *Sexual Health*.

[17] Yitbarek G. Y. (2019). Prevalence of syphilis among pregnant women attending antenatal care clinic, Sede Muja District, South Gondar, Northwest Ethiopia. *Journal of Pregnancy*.

[18] Lendado T. A., Tekle T., Dawit D. (2022). Determinants of syphilis infection among pregnant women attending antenatal care in hospitals of Wolaita zone, Southern Ethiopia. *PLoS One*.

[19] Araújo M. A. L., de Freitas S. C. R., de Moura H. J., Gondim A. P. S., da Silva R. M. (2013). Prevalence and factors associated with syphilis in parturient women in Northeast, Brazil. *BMC Public Health*.

[20] Kelbert G., Freire D. A., Fernandes H. R. (2016). Syphilis screening during prenatal development: missed opportunities in a public maternity hospital in Recife, Brazil. *Brazilian Journal of Sexually Transmitted Diseases*.

[21] Ojo O. C., Arno J. N., Tao G., Patel C. G., Dixon B. E. (2021). Syphilis testing adherence among women with livebirth deliveries: Indianapolis 2014-2016. *BMC Pregnancy and Childbirth*.

[22] World Health Organization (2017). *WHO Guideline on Syphilis Screening and Treatment for Pregnant Women*.

